# Prevalence and impact of comorbid mental disorders in hospitalized patients with obstructive sleep apnea: a protocol for a nationwide retrospective study

**DOI:** 10.1192/j.eurpsy.2024.1497

**Published:** 2024-08-27

**Authors:** D. Nora, A. Freitas, L. Fernandes, A. R. Ferreira

**Affiliations:** ^1^Faculty of Medicine, University of Porto, Porto, Portugal; ^2^CINTESIS@RISE, Department of Community Medicine, Information and Health Decision Sciences (MEDCIDS); ^3^CINTESIS@RISE, Department of Clinical Neurosciences and Mental Health, Faculty of Medicine, University of Porto; ^4^Psychiatry Service, Centro Hospitalar Universitário de São João, Porto, Portugal

## Abstract

**Introduction:**

Obstructive sleep apnea (OSA) is a common sleep disorder in the adult population, often associated with an increased prevalence of comorbid conditions such as obesity and diabetes, but also several mental disorders that have been independently associated with worse hospitalization outcomes in a variety of situations. However, and despite such associations, there is a relative dearth of studies exploring comorbid psychopathology beyond depression and anxiety, and no studies seem to address the impact of comorbid mental disorders on the hospitalization outcomes of patients with OSA.

**Objectives:**

This study aims to characterize and compare mental comorbidities among hospitalization episodes of adult patients with and without OSA held in mainland Portugal, regardless of the primary cause of admission, and to analyze the impact of such comorbidities on hospitalization outcomes.

**Methods:**

An observational retrospective study will be conducted using an administrative database comprising de-identified routinely collected discharge data from all Portuguese mainland public hospitals. Inpatient episodes spanning from 2008 to 2015 will be categorized into two groups according to the presence of an OSA code (ICD-9-CM codes 780.51, 780.53, 780.57, 327.20 and 327.23). For both groups, mental disorders will be identified according to categories 650 to 670 of the Clinical Classifications Software (CCS) for ICD-9-CM. Descriptive, univariate, and multivariate analyses will be performed. Study reporting will comply with the RECORD statement guidelines.

**Results:**

Out of 6,072,538 sampled episodes, 57,301 have an OSA code. Prevalence of any comorbid mental disorder is 30.4% in the OSA group, and 19.3% in the non-OSA group. For both groups, sociodemographic, administrative, and clinical variables will be characterized and compared, as well as the prevalence of each mental disorder category, yearly hospitalization trends, and most common primary diagnoses. Hospitalization outcomes, including length of stay, in-hospital mortality, and readmissions, will be compared taking into consideration the presence of CCS categories of mental disorders.

**Conclusions:**

We expect to improve the understanding of the prevalence of mental comorbidities among hospitalized patients with OSA, including understudied mental disorders, and to elucidate their impact on relevant hospitalization outcomes, thus highlighting the need to recognize and treat this common association to achieve optimal outcomes.

**Disclosure of Interest:**

None Declared

